# MERTIL *for Parents*: Pilot Study of an Attachment and Trauma-Informed Online Parenting Program

**DOI:** 10.3390/children13010007

**Published:** 2025-12-19

**Authors:** Zoe C. G. Cloud, Jessica E. Opie, Nicole Paterson, Anne-Marie Maxwell, Anna T. Booth, Holly Foster, Ellen T. Welsh, Tanudja Gibson, Shikkiah de Quadros-Wander, Jennifer E. McIntosh

**Affiliations:** 1 The Bouverie Centre, La Trobe University, Melbourne, VIC 3056, Australia; jo515@cam.ac.uk (J.E.O.); n.paterson@latrobe.edu.au (N.P.); a.booth@latrobe.edu.au (A.T.B.); h.foster@latrobe.edu.au (H.F.); e.welsh@latrobe.edu.au (E.T.W.); t.gibson@latrobe.edu.au (T.G.); s.dequadros-wander@latrobe.edu.au (S.d.Q.-W.); jenn.mcintosh@latrobe.edu.au (J.E.M.); 2 Department of Public Health and Primary Care, University of Cambridge, Cambridge CB2 0SR, UK; 3 Tresillian Family Care Centres, Belmore, NSW 2192, Australia; annemarie.maxwell@health.nsw.gov.au; 4 School of Psychological Sciences, Faculty of Medicine, Health and Human Sciences, Macquarie University, Sydney, NSW 2000, Australia; 5 Werribee Mother Baby Service, Mercy Mental Health, Melbourne, VIC 3030, Australia

**Keywords:** early relational health, parent–child relationships, attachment, early intervention

## Abstract

**Highlights:**

**What are the main findings?**

• MERTIL *for Parents*, a brief online attachment and trauma-informed program, was highly acceptable to parents who engaged with the intervention, with most reporting the content was useful, the length appropriate, and all were willing to recommend it.
• At three-month follow-up, parents reported significant improvements in parenting confidence, parent–child attunement, enjoyment of the relationship, help-seeking amenability, and reduced irritability, with medium-to-large effect sizes.

**What is the implication of the main finding?**

• MERTIL *for Parents* shows promise as a scalable intervention to support early relational health in families of young children. The relational and attachment-based content fills an important gap in the current online parenting program market, where brief, accessible, and evidence-informed relational interventions are scarce.
• These findings support further research on long-term outcomes and broader implementation across diverse family and service contexts.

**Abstract:**

**Background:** Early relational health is critical for childhood development, and disruptions in infant-caregiver trust can heighten risk. MERTIL *for Parents* is a brief, universal, online program designed to build parental understanding of relational trust and its role in infant development. This pilot study is the first evaluation of the program. **Objectives:** To evaluate the acceptability and short-term outcomes of MERTIL *for Parents*. **Methods:** Seventy-three parents (*n* = 69 mothers) of children aged 0–5 years, referred by practitioners, completed the program and online questionnaires at baseline (*N* = 73), post-intervention (*n* = 50), and three-month follow-up (*n* = 25). **Results:** The program demonstrated high acceptability: 98% of parents who engaged with the program found the content useful, 92% felt the length was appropriate, and all would recommend it to others. Program completion and satisfaction did not vary by sociodemographic or psychosocial characteristics. At three-month follow-up, parents reported significant improvements in their enjoyment of the parent–child relationship (*p* < 0.001), attunement to their child (*p* < 0.001), parenting confidence/competence (*p* = 0.004 and *p* = 0.003), help-seeking amenability (*p* < 0.001), and a reduction in irritability toward the child (*p* < 0.001), with medium to large effect sizes. No significant changes were observed for items assessing reflective functioning or perceived rejection. **Conclusions:** MERTIL *for Parents* shows strong acceptability and promising preliminary outcomes as a scalable, attachment and trauma-informed intervention supporting early relational health. Future research should examine long-term impacts and evaluate broader implementation across diverse families and service contexts.

## 1. Introduction

Relational health within the infant-caregiver dyad is shaped by the parent’s ability to provide security, responsiveness, and sensitivity, all of which help the infant to develop trust and are fundamental to the child’s optimal socioemotional growth [1,2]. Conversely, relational trauma involves continuous and accumulating disturbances to the child’s early care environment (e.g., neglect, maltreatment, insufficient or inconsistent emotional care). As children develop in a relational context, it is the everyday interactional patterns with their caregiver that have been shown to impact child brain development, self-regulatory capacity, and later risk of developing psychopathology [2,3,4]. Thus, relational wellbeing or trauma, respectively, can form the foundation of either a resilient or at-risk trajectory. Importantly, elements of relational health, such as parental sensitivity and the quality of attachment, are modifiable [5,6,7]. With this knowledge, it is critical to promote positive caregiving via timely interventions like psychoeducation that increase parenting capacity, enabling parents to promote child development and afford their child the best start in life.

### 1.1. Program Rationale: Brief, Universal and Accessible Support for Early Relational Health

In 2018, over 1700 Australian Maternal Child Health (MCH) nurses participated in MERTIL (My Early Relational Trauma-Informed Learning) for Practitioners, a 14 h self-directed, interactive, professional development program to enhance practitioners’ ability to identify and prevent relational trauma. Evaluation of the program revealed improved practitioner competence and confidence in recognising relational trauma [8]. However, capacity to respond to identified early relational trauma was inhibited by inadequate referral options for parents, particularly in rural and remote settings.

Currently, few, brief, universal, psychoeducational self-help programs exist that are both accessible to parents and explicitly target relational health in the early years [9]. Most available programs are either in-depth, indicated interventions (e.g., Parent–Child Psychotherapy [10,11]), targeted at parents of children aged over 2-years (e.g., Triple P Brief Online [12], CONNECT Program [13]), or focus narrowly on behavioural concerns such as sleep or feeding [14], overlooking the centrality of early relational health.

Universal parenting programs, by contrast, are available to all interested parents regardless of risk status, thereby supporting prevention, normalising parenting challenges, and avoiding stigma [15,16]. Such programs are typically more cost-effective, scalable, and easier to disseminate broadly [15], and have been associated with improved parental self-efficacy, mental wellbeing and child socioemotional outcomes [16]. Yet, those that focus on early relational health (e.g., Circle of Security [17]), are generally group-based and delivered over multiple sessions, creating accessibility barriers for many families [18,19].

Digital delivery of universal parenting interventions addresses many of these limitations by providing self-directed content that is flexible, private, and scalable. Meta-analyses indicate that online parenting programs can enhance parental knowledge, efficacy, and confidence, while reducing parental mental health problems (e.g., anxiety and anger) and negative parenting practices [20,21]. At the child level, decreases in emotional and behavioural problems have been observed [22], and at the relational level, such programs have been successful in reducing negative parent–child interactions [16]. The flexibility and accessibility of online delivery also reduce stigma and logistical barriers to participation, particularly for families in regional or remote areas [23].

### 1.2. Program Content: MERTIL for Parents

MERTIL *for Parents* (My Early Relational Trust-Informed Learning) was developed to help fill the aforementioned referral gap highlighted by practitioners. It is informed by attachment theory [24] and developmental neuroscience [2,25], emphasising the importance of secure infant-caregiver bonds for lifelong socioemotional wellbeing [4,26,27]. In line with attachment principles [24], MERTIL *for Parents* emphasises parental sensitivity, responsiveness and reflective capacity as key mechanisms for promoting secure attachment. Parents are introduced to concepts such as “safe base” caregiving, recognising and responding to infant cues, and fostering trust through predictable and nurturing interactions. Further, the program applies a trauma-informed lens by recognising that many parents have themselves experienced adversity which may impact their caregiving beliefs and behaviours [28]. Thus, MERTIL for Parents adopts a strengths-based, non-stigmatising approach that validates parents’ struggles and emphasises safety. Program content avoids pathologising language, instead encouraging parents to make small, achievable changes that build relational trust. Animated modules model calm, supportive interactions and highlight the impact of stress and trauma on both parent and child nervous systems, drawing on developmental neuroscience to explain how relational safety can buffer adversity [27]. By integrating trauma-informed practices, the program aims to reduce shame, enhance parental self-compassion and help-seeking amenability, and support parents to regulate their own emotional responses in caregiving contexts.

A key feature of MERTIL *for Parents* is its co-design approach. The program was developed collaboratively with parent consumers, early childhood clinicians, First Nations practitioners and maternal and child health nurses, ensuring contextual and cultural relevance. Co-design processes enhance engagement, program credibility, and real-world applicability [29]. A multiphase feedback process, involving researchers, clinicians, and a production team, ensured that the content was engaging, memorable, and conducive to successful program completion. The final resource consists of four concise, animated 10 min chapters with downloadable resources and tip sheets. This format was intentionally designed to reduce dropout and accommodate parents’ time constraints, while remaining engaging, informative, and accessible.

By equipping parents with relational knowledge and skills grounded in attachment and trauma-informed frameworks, MERTIL *for Parents* offers a preventative early mental health resource with potential public health impact. As a universal, online, strengths-based program, it may help mitigate emerging relational difficulties before they reach clinical thresholds, thereby reducing the longer-term burdens on child mental health and social service systems. However, despite the promise of such universal, self-directed online parenting programs, acceptability and effectiveness must be established before widespread dissemination.

### 1.3. The Present Study

This pilot study had two primary aims: to evaluate both the acceptability and the preliminary effectiveness of the MERTIL *for Parents* program. Given the exploratory nature of this pilot, outcomes are intended to provide early evidence of promise and guide future program refinement.

#### 1.3.1. Acceptability (Aim 1)

Acceptability, defined as stakeholders’ perception of a treatment or intervention as satisfactory, appropriate, or agreeable [30], is a key determinant of intervention uptake and reach. This is particularly relevant in digital health contexts, where acceptability influences engagement and outcomes [31,32]. Acceptability was assessed via three metrics: (1) parent completion rates; (2) parent-reported satisfaction at program-exit (ratings of content usefulness, program length and likelihood of recommending the program), and (3) the influence of sociodemographic and psychosocial factors (e.g., parent age, gender, education, first-time parent status, financial difficulties, trauma history, anxiety/depressive symptoms) on completion and satisfaction. Given the high degree of parent and practitioner co-design in program development, it was hypothesised that the program would demonstrate strong acceptability, with minimal variation across parent sociodemographic and psychosocial factors.

#### 1.3.2. Preliminary Effectiveness (Aim 2)

The second aim was to explore whether MERTIL *for Parents* was associated with short-term changes in caregiving outcomes consistent with attachment and trauma-informed foundations. Outcomes of interest were parental perceptions of the parent–child relationship (e.g., enjoyment, warmth, attunement), parenting confidence and competence, parental reflective functioning, irritability toward the child, and willingness to seek professional support, assessed via changes in parent self-report items. It was hypothesised that parents would demonstrate improvements in all caregiving outcomes at three-month follow-up.

## 2. Materials and Methods

### 2.1. Participants

Participating parents were a sample of 73 parents who provided consent, completed the baseline questionnaire, and met the following inclusion criteria: (a) parent or guardian of a child aged 0–5 years or currently pregnant; (b) program was recommended by a practitioner from a participating pilot site, (c) aged 18 years or older, (d) residing in Australia, and (e) able to complete questionnaires and program materials in English. Of the 73 parents enrolled at baseline (Time 1), the majority identified as female (*n* = 69; 94.5%), with 74.0% serving as the primary caregiver (*n* = 54) and 17.8% sharing caregiving responsibilities equally with another parent or carer (*n* = 13). Four fathers (5.5%) participated in the study; of these, two were primary caregivers, one shared caregiving equally, and one was not a primary caregiver. Most parents were aged 26–35 years (*n* = 48; 65.8%), followed by 36–45 years (*n* = 22; 30.1%), with a small number aged 19–25 years (*n* = 3; 4.1%). The majority of parents had one child (*n* = 50; 68.5%) or two children (*n* = 18; 24.7%). Nearly all parents spoke English at home (*n* = 72; 98.6%) and were in a relationship with their child’s other biological parent (*n* = 65; 89.0%). Most parents had completed high school (97.3%), with a large proportion holding qualifications beyond high school, including TAFE/college diplomas (*n* = 15; 20.5%), undergraduate degrees (*n* = 29; 39.7%), or postgraduate degrees (*n* = 27; 37%). Regarding employment, approximately one-third were full-time stay-at-home parents (*n* = 25; 34.2%), one-third were engaged in full-time paid work (*n* = 24; 32.9%), and one-third worked part-time (*n* = 26; 35.6%). A small number were studying (*n* = 2; 2.7%) or unemployed (*n* = 5; 6.8%). Financially, most parents described their situation as “okay” (*n* = 58; 79.5%) or “well off” (*n* = 14; 19.2%), while one parent (1.4%) reported they were “not getting by”.

### 2.2. Measures

Parents completed online questionnaires at three time-points: baseline/pre-intervention (Time 1); immediately following completion of the program (Time 2); and three months after completion (Time 3). Baseline measures assessed sociodemographic and psychosocial characteristics, and caregiving outcomes. Post-intervention measures assessed program acceptability and satisfaction, and follow-up measures included repeated items from baseline to evaluate change in key outcomes.

#### 2.2.1. Sociodemographic Questionnaire (Time 1)

At baseline, parents completed a 17-item questionnaire assessing demographics (8 items: gender, postcode, age, cultural background, language spoken, education, work/study status, and financial hardship), and family composition (5 items: relationship status, parental/guardianship status, relationship to the child, and number of children in the household). Further, 4 items assessed psychosocial functioning, including history of psychological trauma (dichotomous yes or no: *“have you experienced any serious personal trauma in your life? That is, something that happened to you or your family in the past or recently, that felt frightening or overwhelming, and interfered with your ability to function well in life for more than a few days?”*), the impact of trauma on parenting (1-item rated on a 7-point Likert scale, ranging from 1 = not at all to 6 = almost always), and anxiety and depressive symptoms (2-items rated on a Likert scale ranging from 1 = not at all to 4 = nearly every day).

#### 2.2.2. Repeated-Measures Questionnaire (Time 1 and Time 3)

Key parenting outcomes were assessed at baseline and three-month follow-up using a combination of items from validated scales, items adapted from established Australian longitudinal cohort studies, and one study-generated item. To reduce participant burden, the three-month follow-up survey was shortened, retaining only the core outcome items needed to assess parental perceptions of the parent–child relationship (e.g., enjoyment, warmth, attunement), parenting confidence and competence, parental reflective functioning, irritability toward the child, and willingness to seek professional support (see Table 3). Full descriptions of the broader item pool are published elsewhere [33].

#### 2.2.3. Post-Intervention Questionnaire (Time 2)

Immediately following completion of MERTIL *for Parents*, parents were prompted to complete 3-items embedded within the online platform to assess program usefulness (e.g., “The MERTIL *for Parents* program content was useful to me as a parent”) and satisfaction (e.g., “The length of the program was about right”) on a 4-point Likert scale from strongly disagree (1) to strongly agree (4). Parents were also asked whether they would recommend the program to other parents (0 = No, I wouldn’t recommend; 1 = Yes, I would recommend to some parents; 2 = Yes, I would recommend to all parents).

### 2.3. Procedure

Based on pre-existing professional networks, several publicly funded child, family and maternal health centres across New South Wales (Australia) were chosen as pilot sites for parent recruitment. Pilot site principal investigators invited interested practitioners to contact the research team with their name, email, and pilot site details. Participating practitioners were provided with free access to MERTIL *for Practitioners* online training [8]. The research team enrolled them in the study through the online platform, where they were provided with a consent form to review and complete before attending an information session with background training on MERTIL *for Parents* (120 min web-based information and training session on MERTIL for Parents, conducted by authors JM and JO). The information session included: (a) an overview of the study; (b) a viewing of MERTIL *for Parents*; and (c) a refresher on signs and symptoms of early relational trauma. Practitioners were provided with a companion handbook, which included background information on the program, development and co-design processes, copies of all non-video program resources, references to relevant publications and copies of the parent questionnaires. A total of 47 practitioners across 8 metropolitan, regional, and rural pilot sites were then invited to begin recommending the MERTIL *for Parents* program to parents during their in-person or telehealth contact visits during the parent recruitment period (March 2024–July 2024).

After being recommended the program by their practitioner, interested parents completed an online expression of interest and were subsequently provided with consent materials and enrolled in the online program by the research team. Participants were granted free access to the program and downloadable support materials for two months following completion of consent. Parent participants completed questionnaires at three time-points: prior to commencing the program (baseline; Time 1), immediately following the program completion (post-intervention; Time 2), and at three months following program completion (Time 3). Upon completion, parents received a personalised certificate of completion. A detailed description of the recruitment procedure and research protocol has been published elsewhere [33]. The study was approved by La Trobe University’s Human Research Ethics Committee (HREC: 22096) and Sydney Local Health District (RPAH) Human Research Ethics Committee (X22-0286 & 2022/ETH01680). Before consenting, all parents received written information about the study and were offered the opportunity to have questions answered.

#### 2.3.1. Intervention

As an online program, MERTIL *for Parents* is accessed via the internet and can be viewed on any online platform (i.e., phone, computer, or tablet). The primary component of MERTIL *for Parents* comprises 40 min of pre-recorded videos, consisting of four ‘chapters’, with each chapter approximately ten minutes in duration. Chapter one (“Welcome to MERTIL *for Parents*”) introduces the program, explains the concept of relational trust, its significance, and outlines who might benefit from the program. It also provides self-care tips for parents engaging with the content. Chapter two (“Why trust matters”) explores the role of attachment and trust in parenting, particularly when a parent has experienced relational trauma. It addresses the realistic expectations for parents and children, debunks myths about babies (e.g., babies should be happy all the time; babies become ‘spoilt’ by too much attention), introduces the concept of the “good enough” parent, and offers practical strategies for fostering trust and shared delight within the parent–child relationship (e.g., cuddling, smiling, singing/dancing together). Chapter three (“Trust and trauma; rupture and repair”) focuses more on building relational trust through parent predictability, the process of rupture and repair, and understanding the distinction between good stress (i.e., novelty and developmentally appropriate challenge) and bad stress (i.e., feeling unsafe; stress that occurs too often or for too long). It examines the impact of parental trauma and family violence on parenting and infant development and introduces the parent and child steps of ‘the trust dance’, a framework for nurturing securely attached relationships. The final chapter (Chapter four: “Becoming your best parent, with support”) summarises the program, emphasises the importance of seeking and utilising support, and provides guidance on help-seeking behaviours, including recognising when professional help may be needed.

The program’s evidence-based narrative was carefully designed to be engaging and accessible to a diverse parent audience. Moments of humour are incorporated to balance the occasional heavy content, while lay language is used to effectively communicate key messages. The program emphasises trust, safety, relatability, and acceptance, with a narrator’s voice that is intentionally warm, calm, non-blaming, and reassuring to foster trust and engagement. Along with the video content, secondary program components are provided, including downloadable parent tipsheets, posters, and professional support contacts.

#### 2.3.2. Analytic Plan

Data were downloaded as spreadsheet files and imported into IBM SPSS Statistics (Version 30) to generate descriptive statistics and analyse quantitative data. Aim 1 (acceptability) was assessed by examining parent completion rates based on dropout between study time-points. Satisfaction with program usefulness, length and likelihood of recommending the program were summarised using descriptive statistics from post-intervention survey data (Time 2). To examine whether baseline characteristics influenced parent satisfaction with the program, a composite satisfaction score was computed based on the average of the parent-report responses regarding program usefulness and length. Then, overall satisfaction score was compared across participant subgroups defined by categorical baseline variables. Independent-samples *t*-tests were conducted when the predictor variable had two levels (e.g., gender), and one-way analysis of variance (ANOVA) was used when the predictor had three or more levels (e.g., education level, language spoken at home).

For Aim 2, preliminary efficacy of the MERTIL *for Parents* program was evaluated by examining paired samples *t*-tests to explore within-person item-level changes from baseline (Time 1) to 3-month follow-up (Time 3) across items related to parental perceptions of the parent–child relationship (e.g., enjoyment, warmth, attunement, and perceived rejection), parenting confidence and competence, parental reflective functioning, irritability toward the child, and willingness to seek professional support. Given that 12 paired sample *t*-tests were conducted, we applied a Bonferroni correction to reduce the likelihood of Type I error. The corrected significance level was calculated as 0.05/12 = 0.004. Accordingly, only *p*-values equal to or below 0.004 were considered statistically significant. Adjusted *p*-values are presented in Table 3. Effect sizes were calculated to estimate the magnitude of observed effects and interpreted according to conventional thresholds (Cohen’s d; small = 0.20; medium = 0.50; large = 0.80 [34]).

## 3. Results

### 3.1. Aim 1: Acceptability

Parent acceptability of the program was indicated via completion rates and parent satisfaction at program exit.

#### 3.1.1. Parent Completion Rates

**Figure 1:**
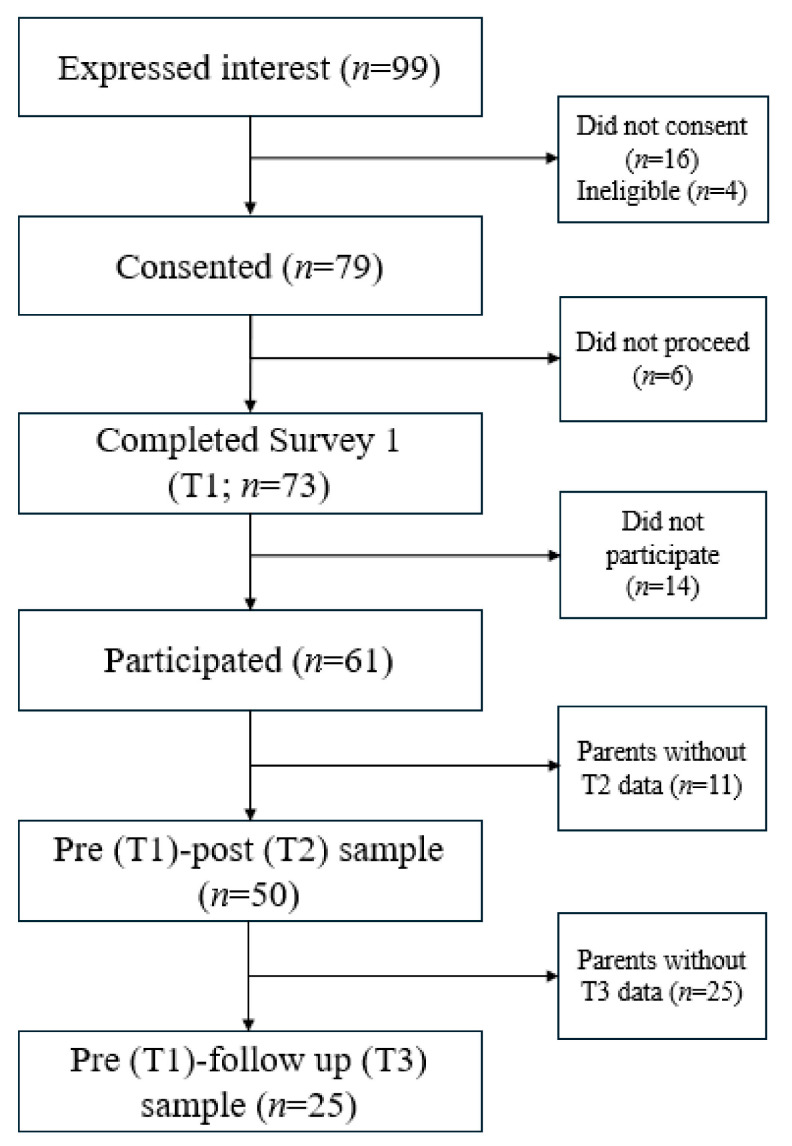
Flow Diagram of Study Participants.

Over a five-month recruitment period (March–July 2024), 99 parents initiated registration for MERTIL *for Parents* and were screened for eligibility. Of these, 79 met inclusion criteria, consented to participate, and were provided access to the program. Participant engagement and completion were monitored for approximately eight months, concluding in October 2024. During this time, 61 parents completed the full intervention, representing 77.2% of those who consented and 83.6% of parents who commenced the program (defined as completing at least one video module). On average, participants completed the program in 9.85 days (SD = 12.73; min = 1 day, max = 15 days). At the three-month follow-up, outcome data were available from 41.0% of program completers. The study flow is depicted in Figure 1.

Next, baseline sociodemographic and psychosocial variables were examined to assess whether any factors were associated with program completion or follow-up participation. As seen in Figure 1, of the 79 parents who consented at baseline (Time 1), 50 completed the program and the post-intervention questionnaire (Time 2), and a subsample of 25 completed the three-month follow-up (Time 3). Chi-square analyses were conducted to compare participants across time points. No significant differences were found between those who did not complete the program (Time 1 only), those lost to follow-up (Times 1 and 2 only), or those retained at all time-points (Times 1–3) based on age (χ^2^(1, 73) = 2.29, *p* = 0.797, *V* = 0.18), gender (χ^2^(1, 73) = 2.00, *p* = 0.540, φ = 0.15), education level (χ^2^(1, 73) = 10.15, *p* = 0.088, *V* = 0.38), number of children (χ^2^(1, 73) = 5.82, *p* = 0.319, *V* = 0.29), financial adversity (χ^2^(1, 73) = 3.39, *p* = 0.139, *V* = 0.22). Although First Nations identity and home language were assessed at baseline, these variables could not be included in subgroup analyses due to very low cell counts (*n* = 1 for First Nations identity; *n* = 0 for participants who did not speak English at home).

Regarding baseline psychosocial characteristics, 35.6% of parents (*n* = 26) reported a history of serious personal trauma, and 21.9% (*n* = 16) indicated that their trauma history affected their parenting at least some of the time. Additionally, 56.2% (*n* = 41) of parents reported experiencing anxiety and 42.5% (*n* = 31) reported experiencing depressive symptoms at least some of the time. Importantly, there were no significant differences in program completion rates based on baseline parents’ history of trauma (χ^2^(1, 73) = 4.36, *p* = 0.139, φ = 0.24), perceived impact of trauma on parenting (χ^2^(1, 73) = 2.11, *p* = 0.431, φ = 0.21), or self-reported symptoms of anxiety (χ^2^(6, 73) = 4.61, *p* = 0.595, φ = 0.18) or depression (χ^2^(6, 73) = 3.52, *p* = 0.742, φ = 0.16).

#### 3.1.2. Parent Satisfaction with MERTIL for Parents

At program-exit, 50 parents responded to three Likert-scale items indicating their overall level of satisfaction with the MERTIL *for Parents* program (Table 1).

As seen in Table 1, parent responses indicated a high level of satisfaction with the program, with 98% of parents either agreeing or strongly agreeing that the content was useful, and 92% of parents agreeing or strongly agreeing that the length of the program was appropriate. Further, all parents reported that they would recommend the program to others, although a small proportion suggested they would recommend it selectively (14%).

To further explore parent acceptability, the association between baseline characteristics and overall program satisfaction was examined using independent-samples *t*-tests (for categorical variables with two levels) or one-way ANOVA (for categorical variables with three or more levels). There were no significant differences in program satisfaction based on baseline sociodemographic or psychosocial characteristics (Table 2).

### 3.2. Aim 2: Preliminary Effectiveness

The short-term effectiveness of the MERTIL *for Parents* program was examined in the subset of parents who completed the survey at three-month follow-up (*n* = 25). Outcomes included parental perceptions of the parent–child relationship (e.g., enjoyment, warmth, attunement, and perceived rejection), parenting confidence and competence, parental reflective functioning, irritability toward the child, and willingness to seek professional support. Item-level changes from baseline (Time 1) to follow-up (Time 3) were evaluated using paired-samples *t*-tests, with effect sizes (Cohen’s d) reported to indicate the magnitude of observed changes (Table 3).

**Table 3:** Changes in Parenting Outcomes from Baseline (Time 1) to 3-Month Follow-Up (Time 3; *N* = 25).

Measure [Citation]	Item	Possible Range	Time 1, M (SD)	Time 3, M (SD)	*p*	*d*
Maternal Postnatal Attachment Scale [35]	*1. When I am with my child, I get enjoyment and satisfaction.*	1–4, higher scores indicate a stronger attachment relationship.	1.72 (0.46)	3.76 (0.44)	<0.001	0.68
	*2. When I interact with my child, I feel confident and competent.*		3.17 (0.49)	3.52 (0.51)	0.004	0.57
Perinatal Emotional Growth Index [36]	*1. I can read my child’s signals and know what she/he needs or wants*	1–5, with higher scores indicate a greater level of agreement.	3.96 (0.54)	4.44 (0.51)	<0.001	0.59
Parental Reflective Functioning Questionnaire [37]	*1. I try to understand the reasons why my child misbehaves*	1–7, with higher scores indicating a greater level of agreement.	6.04 (1.50)	6.00 (1.47)	0.894	0.03
	*2. I am often curious to find out how my child feels*		6.44 (0.96)	6.64 (0.86)	0.307	0.21
	*3. I try to see situations through the eyes of my child*		6.00 (1.38)	6.28 (0.94)	0.283	0.22
Items adapted from the Longitudinal Study of Australian Children [38,39]	*Over the past few weeks, how often would you say…*	1–5, with higher scores indicating a greater level of agreement.				
	*1. you showed affection by hugging, kissing and holding your child*		4.88 (0.07)	4.92 (0.06)	0.574	0.11
	*2. you had warm, close times together with your child*		4.72 (0.54)	4.76 (0.44)	0.714	0.08
	*3. you felt your child got on your nerves when he/she cried*		2.52 (0.87)	2.00 (0.80)	<0.001	0.65
	*4. Sometimes, I feel my child does not like me and does not want to be close to me*		4.44 (0.82)	4.68 (0.48)	0.083	0.36
	*5. Overall, as a parent, do you feel that you are…*	1 = having some trouble being a parent; 2 = an average parent; 3 = a better than average parent; 4 = a very good parent.	2.56 (0.92)	3.04 (0.79)	0.003	0.67
Study-specific	*1. I feel confident about seeking parenting or mental health support if I or my child needed it*	1 = No; 2 = Maybe; and 3 = Yes	1.80 (0.50)	2.84 (0.37)	<0.001	2.29

Note. Bonferroni-corrected significance threshold of *p* < 0.004 (0.05/12) was applied.

As shown in Table 3, statistically significant improvements were observed between Time 1 and Time 3 on six-outcomes: enjoyment in the parent–child relationship (*p* < 0.001), attunement to the child (*p* < 0.001), two items assessing parenting confidence/competence (*p* = 0.004 and *p* = 0.003), help-seeking amenability (*p* < 0.001), and irritability toward the child (*p* < 0.001). Effect sizes for these changes were in the medium to large range (*d* = 0.57–2.29). No significant changes were detected for parental reflective functioning (three items), affection, warm close times, or perceived rejection (“child does not like me”).

## 4. Discussion

The present study evaluated the acceptability and preliminary effectiveness of MERTIL *for Parents*, a brief, online, relationally focused parenting program co-designed with maternal and child health nurses, infant mental health specialists, and parents. Specifically, this pilot study examined: (1) parent acceptability of the program, including whether baseline characteristics predicted program completion or satisfaction, and (2) short-term changes in key caregiving items between baseline and three-month follow-up. Among a small sample of parents, preliminary findings indicate support for the MERTIL *for Parents* program as a highly acceptable and effective parenting program to improve caregiver knowledge of early relational health, and associated improvements in parent confidence and in the parent-infant relationship.

### 4.1. Acceptability

In support of the first hypothesis, parents reported high satisfaction with both the content and length of MERTIL *for Parents*, with nearly all rating it as useful, appropriate in length, and worth recommending. Completion rates were also encouraging: over 80% of parents who commenced the program completed it, typically within 10 days. Importantly, neither completion nor satisfaction varied by sociodemographic or psychosocial characteristics (including parents’ prior experiences of serious psychological trauma, anxiety or depressive symptoms), suggesting broad accessibility and relevance across diverse groups. This level of engagement compares favourably with digital parenting programs more generally, where dropout is often substantial [40]. Nonetheless, only 41% of completers provided data at three-month follow-up, highlighting the need for strategies to support longer-term retention.

Prior evidence on the relationship between program duration and effectiveness is mixed: some studies suggest longer trauma-focused parenting interventions are more effective in improving parent mental health and caregiving-related outcomes [28], others report no clear link [41,42], and some find advantages for briefer formats [43]. Against this backdrop, the strong acceptability and relatively favourable completion rates of a concise program such as MERTIL *for Parents* are promising. While not intended to replace intensive, multi-session models for traumatised parent–child relationships (e.g., Child–Parent Psychotherapy; Lieberman et al., 2020 [11]), its brevity and accessibility position it well as an early, preventative option.

Finally, the absence of differences in satisfaction by baseline characteristics (including parents’ prior experience of psychological trauma, anxiety and depressive symptoms) suggests that the program may be accessible and acceptable to parents with diverse psychosocial backgrounds, including those who are often harder to reach through traditional parenting interventions. The co-design process, involving maternal and child health nurses, infant mental health specialists, and parents themselves, may have strengthened the program’s social validity and reduced stigma, both of which are critical for engagement among vulnerable groups [28,44]. Future research could help identify which design elements most strongly promote acceptability and whether these contribute to sustained engagement across parent subpopulations. Overall, acceptability findings suggest that the short, animated modules of MERTIL *for Parents*, presented in a strengths-based and non-blaming tone, may help to address previously identified barriers to digital parenting programs.

### 4.2. Preliminary Effectiveness

Consistent with the second aim, the findings provide preliminary evidence for the effectiveness of MERTIL *for Parents* among program completers. From baseline to three-month follow-up, parents reported significant improvements in multiple domains, including parenting confidence and competence, enjoyment and attunement in the infant-caregiver relationship, amenability to help seeking, and reduced irritability toward their child. The pattern of improvements aligns with the program’s attachment-based foundations, which emphasise parental sensitivity, responsiveness, and reflective capacity as pathways to stronger relational security. Attachment-focused programs share this goal but differ in how they support change: some directly target parenting behaviours through methods such as live coaching or video feedback [10,11], whereas others seek to shift caregivers’ internal representations, including their reflective functioning [17]. Although MERTIL *for Parents* does not employ active, therapist-led techniques, the program introduces accessible concepts such as “safe base” caregiving, recognising infant cues, identifying all behaviour as communication, and creating predictable, nurturing interaction patterns. This psychoeducational approach may help parents feel more confident, more attuned to their child, and better equipped to manage everyday stressors.

In contrast, no significant improvements were detected in reflective functioning or measures of affection and warmth. This was unexpected given the strong prevalence of these factors as predictors of secure attachment and as moderators of effectiveness in other relationally focused parenting interventions [45,46]. However, this finding likely reflects a ceiling effect, with many parents entering the program already reporting relatively high levels of warmth, closeness, and reflective capacity. Nevertheless, future research using a comparison group is needed to clarify why some parenting behaviours shifted over time while others remained stable, and to determine the degree to which brief, self-directed programs can influence long-term parenting behaviours and reflective capacity.

Despite the lack of significant change in some outcomes, all items trended in the predicted direction. The largest effect size was found in the improvement in parents’ amenability to help-seeking. This may reflect the programs’ trauma-informed and relationally focused design, which normalises parenting challenges and frames support-seeking as a sign of proactive strength. Given that over a third of parents reported a trauma history at baseline, this non-blaming, strengths-based approach could reduce service mistrust and enhance openness to professional help. The brief, self-paced online format may also serve as a psychologically safe entry point, lowering stigma and logistical barriers to accessing services. Importantly, enhanced willingness to seek support represents a valuable early intervention outcome in itself, as it may facilitate earlier identification of difficulties and connection with further care where needed.

Together, findings suggest that even a brief, self-directed online program may foster meaningful changes in parental perceptions and behaviours relevant to early relational trauma. Importantly, preliminary effectiveness findings address a critical gap identified in the literature: the scarcity of brief, evidence-based, universally available parenting programs that explicitly target relational trust and early relational health. By offering universal, strengths-based support, MERTIL *for Parents* may help normalise parenting challenges, reduce stigma, and promote parental self-efficacy and early relational health, positioning it as an important complement to more intensive interventions, particularly in settings where early prevention is underdeveloped.

### 4.3. Strengths, Limitations and Future Research

While this study provides innovative insights into the utility of a brief, online relational program, several limitations should be noted. First, the pilot population was limited to parents located in New South Wales, Australia; generalisability to wider populations remains untested. Second, rates of parent uptake of MERTIL *for Parents* were not explicitly measured; however, anecdotal reports from practitioners indicated lower-than-expected rates of program uptake during the pilot, resulting in a smaller parent sample than anticipated [33]. Thus, study findings reflect outcomes for a population of parents who chose to engage with the program, and we do not know whether or how they differed from parents who were offered the program and didn’t engage. Future studies of MERTIL *for Parents* should measure parent uptake in addition to outcomes, to better understand the potential reach and impact of this and similar digital interventions. Third, only one participant who completed all three time-points identified as First Nations and all participants reported English as the primary language spoken at home. This lack of cultural representation highlights the need for further research to explore whether MERTIL *for Parents* can be appropriately adapted and effectively delivered to culturally and linguistically diverse populations or communities, including those experiencing digital access disadvantage (particularly given the lack of live technical support). Fourth, attrition between baseline (*n* = 75) and follow-up (*n* = 25) was moderate, and although baseline characteristics did not predict completion, the dropout rate highlights challenges common to online interventions [16,19,22,29,40]. Further, all parent data were self-reports, introducing potential biases related to social desirability and recall. Finally, data collection was limited to item-level measures to reduce participant burden and align with the brief and accessible format of the program. While most items were drawn from validated instruments, future research should employ complete validated scales to strengthen construct validity.

Despite these limitations, the study has notable strengths. Importantly, the program was developed in direct response to practitioner feedback highlighting the lack of suitable referral options for families at risk of early relational trauma [8]. The relational and attachment-based content of MERTIL *for Parents* therefore fills an important gap in the current online parenting program market, where brief, accessible, and evidence-informed relational interventions are scarce. Even within a modest sample, there was diversity in several aspects of parental background, including psychosocial characteristics, family composition (first-time versus experienced parents), and geographic spread (recruitment across metropolitan and regional pilot sites). Co-design with maternal and child health nurses, infant mental health experts, and parents further strengthens ecological validity and program relevance. Furthermore, parent satisfaction was consistently positive, and despite the brevity of the program, meaningful improvements were detected across several items central to infant-caregiver relational health.

These strengths highlight the potential for broader dissemination and warrant further evaluation in larger and more diverse samples, including interstate and international cohorts. Incorporating multi-informant methods (e.g., parent and practitioner) would help overcome limitations of self-report data and provide a more robust picture of program impact. In-depth qualitative investigation may also be valuable, particularly for understanding any nuances in how the program is experienced for subpopulations of parents (e.g., trauma-affected parents or those experiencing perinatal distress). Such work could illuminate mechanisms of change and identify ways to refine delivery for families facing additional relational or contextual challenges.

At the same time, it is important to preserve the program’s universal orientation, as broad accessibility helps to normalise parenting challenges, reduce stigma, and support prevention before difficulties become entrenched. Future research should therefore examine both the effectiveness of MERTIL *for Parents* as a universal resource for all parents, and the potential benefits of tailored or augmented approaches for subgroups with specific relational vulnerabilities. Finally, assessing longer-term outcomes will clarify whether the observed short-term gains are sustained over time, thereby informing the program’s potential contribution to public health efforts to strengthen early relational health.

## 5. Conclusions

In sum, this study provides initial evidence for the acceptability and short-term efficacy of MERTIL *for Parents*, a brief, online, relationally focused parenting program. Findings suggest that the program is well-received by caregivers who complete it, broadly acceptable across diverse backgrounds, and capable of producing meaningful improvements in parenting confidence, relational quality, and help-seeking amenability. With further evaluation, MERTIL *for Parents* has potential to make a significant contribution to the suite of brief online parenting interventions available in Australia and beyond.

Program website (https://www.mertil.com.au/).

## Figures and Tables

**Table 1:** Parent Satisfaction with MERTIL *for Parents* at Program Exit (*N* = 50).

Item and Response	*N* (%)
Program content was useful to me as a parent	
Strongly disagree	1 (2)
Disagree	0 (0)
Agree	44 (64)
Strongly Agree	23 (34)
The length of the program was about right	
Strongly disagree	1 (2)
Disagree	3 (6)
Agree	32 (64)
Strongly Agree	14 (28)
I would recommend the program to other parents	
No, I would not recommend	0 (0)
Yes, I would recommend to some parents	7 (14)
Yes, I would recommend at all parents	43 (86)

**Table 2:** Exploratory Analyses of Parent Satisfaction by Baseline Characteristics (*N* = 50).

Baseline Characteristic	Levels (*n*)	*t/F*	*p*	*d/η2*
Parent age (years)	19–25 (2)/26–35 (33)/36–45 (15)	0.26	0.773	0.01
Gender	Male (4)/Female (45)	−0.42	0.680	−0.22
Education	Some high school (1)/completed high school (12)/undergraduate degree (18)/postgraduate degree (19)	0.11	0.956	0.01
First-time parent status	Yes (35)/No (12)	−0.61	0.545	−0.20
Financial adversity	Some (41)/None (9)	0.05	0.821	0.00
History of trauma	Yes (36)/No (14)	0.37	0.716	0.12
Trauma impacting parenting	Yes (14)/No (22)	0.06	0.951	0.02
Anxiety symptoms	Not at all (19)/Several days per week (23)/more than half the week (6)/nearly every day (2)	0.69	0.562	0.04
Depressive symptoms	Not at all (28)/Several days per week (19)/more than half the week (2)/nearly every day (1)	0.44	0.728	0.03

Note. Analyses for First Nations identity and home language could not be computed due to insufficient sample variability.

## Data Availability

The data that support the findings of this study are available on request from the corresponding author, Z.C.G.C. The data are not publicly available due to their containing information that could compromise the privacy of research participants.
